# T cells with high BCL-2 expression induced by venetoclax impact anti-leukemic immunity “graft-versus-leukemia effects”

**DOI:** 10.1038/s41408-024-01064-0

**Published:** 2024-05-14

**Authors:** Joji Nagasaki, Mitsutaka Nishimoto, Hideo Koh, Hiroshi Okamura, Mika Nakamae, Kazuki Sakatoku, Kentaro Ido, Masatomo Kuno, Yosuke Makuuchi, Teruhito Takakuwa, Yasuhiro Nakashima, Masayuki Hino, Hirohisa Nakamae

**Affiliations:** 1 Department of Hematology, Osaka Metropolitan University Graduate School of Medicine, Osaka, Japan; 2 Department of Tumor Microenvironment, Okayama University Graduate School of Medicine, Dentistry and Pharmaceutical Sciences, Okayama, Japan; 3 Department of Preventive Medicine and Environmental Health, Osaka Metropolitan University Graduate School of Medicine, Osaka, Japan; 4 Department of Laboratory Medicine and Medical Informatics, Osaka Metropolitan University, Osaka, Japan

**Keywords:** Acute myeloid leukaemia, Cancer immunotherapy

**TO THE EDITOR:**

BCL-2, a crucial regulator of the mitochondrial apoptotic pathway, maintains myeloblast survival by sequestering the pro-apoptotic protein BAX [1]. Venetoclax (VEN) is an orally bioavailable BH3-mimetic protein that selectively inhibits BCL-2 and has shown promise against acute myeloid leukemia (AML) [2]. The direct cytotoxic effects of VEN are mediated by BCL-2 inhibition, and the subsequent increase in BAX/BAK results in mitochondrial outer membrane permeabilization [3]. Although the activities of VEN on leukemia cells are being elucidated, its immunological impact on anti-leukemic immunity is unknown. Allogeneic hematopoietic cell transplantation (allo-HCT) is a potentially curative treatment for patients with high-risk AML, and eradication of leukemia cells largely relies on anti-leukemic immunity “graft-versus-leukemia effects,” mainly exerted by donor-derived T cells. Some reports suggest that VEN preserves T-cell function, particularly in effector T cells [4, 5]. Therefore, we hypothesized that among patients who experienced relapsed AML after allo-HCT, VEN might effectively eliminate leukemia cells not only through its direct anti-tumor activity but also by preserving anti-leukemic immunity. In this clinical observational study, we explored the efficacy and immunological activity of treatment with VEN and azacitidine (VEN therapy) by analyzing the clinical outcomes and pre- and post-treatment samples of 12 patients who received VEN therapy for relapsed AML after allo-HCT at our institution.

To investigate the prognostic impact of VEN, we initially assessed the clinical outcomes of VEN therapy. Baseline characteristics and treatment trajectories are presented in Table S1 and Fig. 1A. The treatment schedule is described in the Supplementary Methods. In brief, azacitidine (75 mg/m^2^/day for 5 consecutive days) was administered concurrently with VEN. In principle, VEN at 50 or 200 mg/day was administered for 14 days in the first course and for 14 to 28 days in the second and later courses. The median duration of VEN administration per 28-day cycle was 14 days (range 14–28) in the first course and 21 days (range 2–28) in the second and later courses. No exacerbation of graft-versus-host disease was observed after VEN therapy. We compared the clinical outcomes of VEN-treated patients with those of 61 control patients. Overall survival and relapse mortality at one year were significantly better in the VEN therapy group than in the control group (66.7% vs. 27.3%, *P* = 0.021; 33.3% vs. 65.9%, *P* = 0.046, respectively) (Fig. S1a–c). After propensity score matching based on the age, sex, time of relapse from transplantation, disease status at relapse, karyotype risk [6], and blasts in the bone marrow at the start of treatment, the patient characteristics were almost well balanced (Table S2). The area under the curve in the logistic regression model for the propensity score was 0.846. The median survival time in the VEN therapy group was significantly better than that in controls (not reached vs. 80 days, *P* = 0.008) (Fig. 1B), with an adjusted hazard ratio of 0.22 (95% confidence interval: 0.06–0.73, *P* = 0.014). Relapse mortality at one year was also significantly better (Fig. S1d) without non-relapse deaths (Fig. S1e) in the VEN therapy group. Regarding peripheral blood lymphocytes, the number of B cells decreased after VEN therapy, whereas those of CD4^+^ and CD8^+^ T cells were only slightly decreased (Fig. S1f). These data indicate that VEN therapy is clinically effective for relapsed AML after allo-HCT and lowers relapse mortality without elevating non-relapse mortality.

**Fig. 1:**
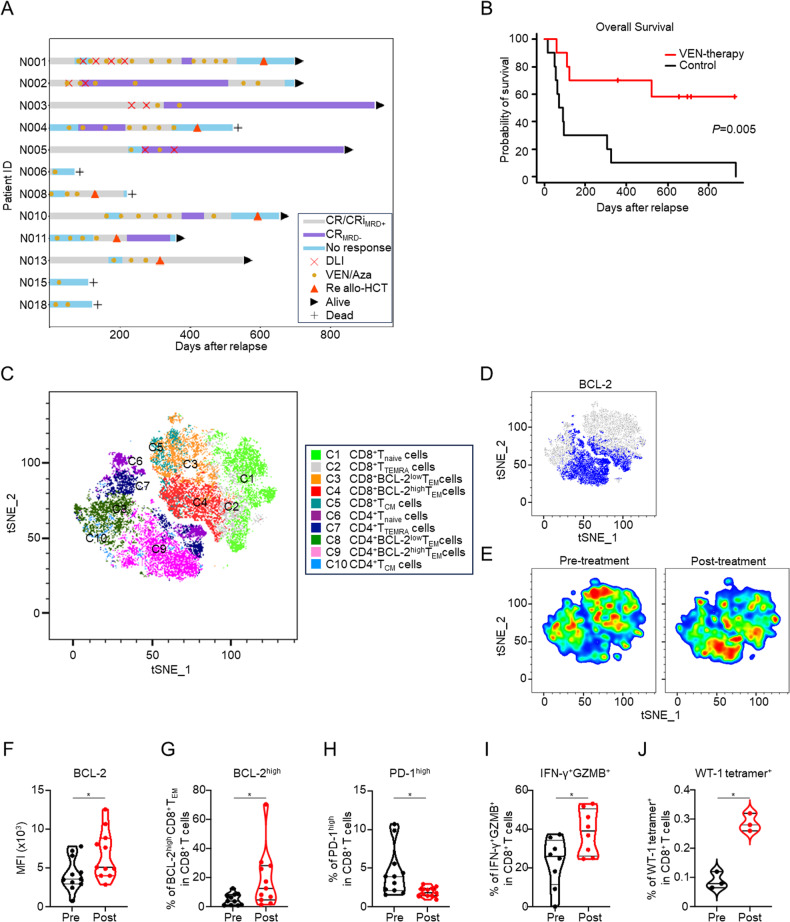
VEN therapy improves the prognosis of relapsed AML after allo-HCT and increases the number of BCL-2^high^ CD8^+^ T_EM_ cells and progenitor/non-exhausted, cytotoxic, and tumor-specific T cells. **A** Swimmer plot of 12 patients who received VEN therapy for relapsed AML after allo-HCT. **B** Comparison of overall survival between propensity score-matched VEN therapy and control groups. **C** Analysis of PBMCs from pre- and post-VEN therapy patients using flow cytometry (*n* = 11 each for pre- and post-VEN therapy patients). T-cell data were extracted, downsized, and concatenated, and a t-SNE plot was generated. Clusters were defined by marker combinations and expression levels. **D** Heatmap depicting the expression level of BCL-2 on the t-SNE map. **E** t-SNE images before and after VEN therapy. Each t-SNE figure is presented separately in density plots. **F** MFI of BCL-2 in CD8^+^ T cells from pre- and post-VEN therapy patients. Summaries of the data are shown (*n* = 11). **G** Proportions of BCL-2^high^ T_EM_ cells in CD8^+^ T cells from pre- and post-VEN therapy patients. Summaries of the data are shown (*n* = 11). **H**–**J** Proportions of PD-1^high^ (*n* = 11) (**H**), IFN-γ^+^GZMB^+^ (*n* = 8) (**I**), and WT-1 tetramer^+^ (*n* = 3) (**J**) cells in whole CD8^+^ T cells. PBMCs from pre- and post-VEN therapy patients were analyzed with flow cytometry. Summaries of the data are shown. Paired two-tailed Student’s *t-*test was utilized for statistical calculations in figures (**F**–**J**); *P < 0.05. ID identification, CR_MRD−_ complete remission without minimal residual disease, CR_MRD+_ complete remission with minimal residual disease, CRi complete remission with incomplete hematologic recovery, DLI donor lymphocyte infusion, Aza azacitidine.

To clarify the impact of VEN therapy on anti-leukemic immunity, we characterized the phenotype of donor-derived T cells using peripheral blood mononuclear cells (PBMCs) from pre- and post-VEN therapy patients. T cells were classified into 10 clusters as follows: first, they were divided into CD4^+^ and CD8^+^ clusters, and each cluster was subdivided into naive T cells (T_naive_; CCR7^+^CD45RA^+^), central memory T cells (T_CM_; CCR7^+^CD45RA^−^), BCL-2^high^ effector memory T cells (BCL-2^high^ T_EM_; CCR7^−^CD45RA^−^BCL2^high^), BCL-2^low^ T_EM_ cells (CCR7^−^CD45RA^−^BCL2^low^), and terminally differentiated effector memory T cells (T_TEMRA_; CCR7^−^CD45RA^+^) (Fig. 1C, D, and S2a). Notably, the BCL-2 expression level in CD8^+^ T cells was significantly higher after VEN therapy than before (Fig. 1E, F, and S2b). Further analysis showed that VEN therapy markedly reduced the number of BCL-2^low^ CD8^+^ T_EM_ cells and increased the number of BCL-2^high^ CD8^**+**^ T_EM_ cells (Fig. 1E, G).

Next, we evaluated the differences in the properties and functions of CD8^+^ T cells before and after VEN therapy. After VEN therapy, there was a lower proportion of exhausted CD8^+^ T cells with high PD-1 expression (Fig. 1H and S2c) and a high proportion of cytotoxic CD8^+^ T cells expressing granzyme B and interferon-γ (IFN-γ) (Fig. 1I and S2d). Moreover, the number of WT-1 tetramer^+^ leukemia-specific CD8^+^ T cells increased following VEN therapy (Fig. 1J and S2e). These results indicate that the functional improvement induced by VEN therapy potentiates the anti-leukemic effects, accompanied by a characteristic change in the population of CD8^+^ T cells and an increase in BCL-2^high^ CD8^+^ T_EM_ cells.

To characterize BCL-2^high^ CD8^+^ T_EM_ cells, we compared their functions with those of BCL-2^low^ CD8^+^ T_EM_ cells in PBMCs or bone marrow samples from post-VEN therapy patients. We first evaluated the association between BCL-2 expression and degree of exhaustion. Among CD8^+^ T_EM_ cells, the proportion of terminally exhausted PD-1^high^ T cells was lower in BCL-2^high^ T cells than in BCL-2^low^ T cells. Thus, BCL-2^high^ T cells suggested progenitor/non-exhausted cells rather than BCL-2^low^ T cells (Fig. 2A). T-cell exhaustion, a state characterized by the hierarchical loss of effector functions and by the expression of multiple inhibitory receptors, is known to diminish the graft-versus-leukemia effect [7]. Next, we assessed the correlation between BCL-2 expression, cytotoxicity, and tumor specificity. BCL-2^high^ CD8^+^ T_EM_ cells secreted more granzyme B and IFN-γ, and contained a higher proportion of tumor-specific T cells than BCL-2^low^ CD8^+^ T_EM_ cells (Fig. 2B, C). Thus, BCL-2^high^ CD8^+^ T_EM_ cells are strongly cytotoxic against and highly specific for leukemia cells. These results indicate that the increase in BCL-2^high^ CD8^+^ T_EM_ cells may increase graft-versus-leukemia activity by enhancing anti-leukemic immunity.

**Fig. 2:**
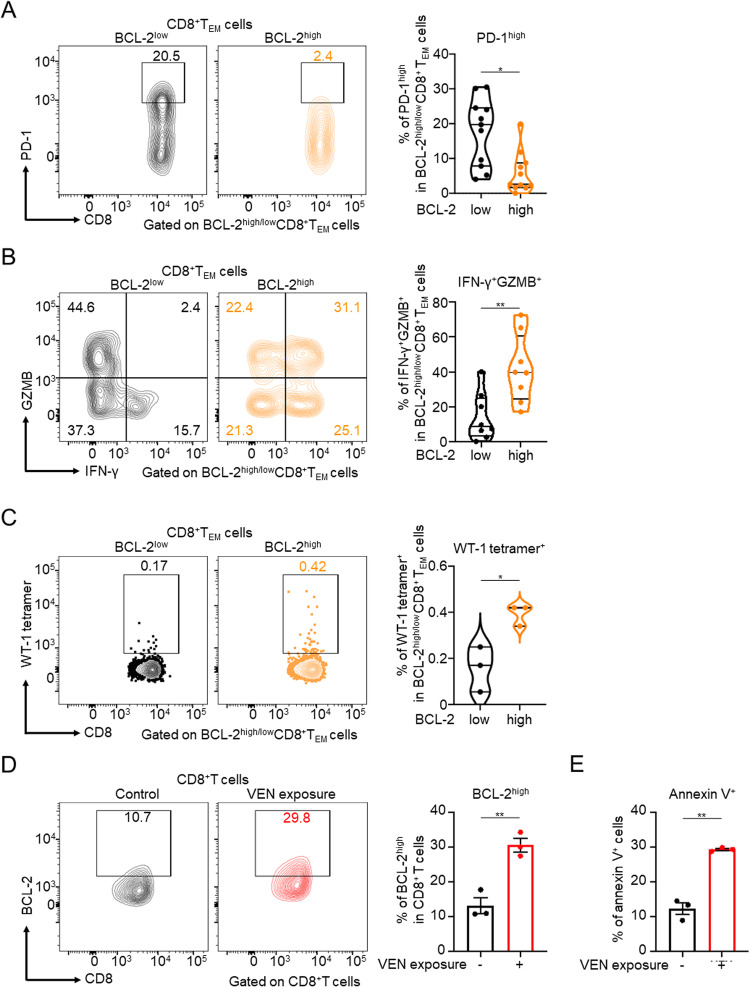
BCL-2^high^ CD8^+^ T_EM_ cells have a progenitor/non-exhausted, cytotoxic, and tumor-specific phenotype, and VEN exposure enhances the anti-tumor immune response of CD8^+^ T cells. **A**–**C** Proportion of PD-1^high^ (*n* = 11) (**A**), IFN-γ^+^GZMB^+^ (*n* = 8) (**B**), and WT-1 tetramer^+^ (*n* = 3) (**C**) cells in BCL-2^high/low^ CD8^+^ T_EM_ cells. Samples were obtained from post-VEN therapy patients, and flow cytometry analysis was performed on PBMCs for PD-1 and WT-1 data, and on bone marrow samples for cytokine data. **D** Proportion of BCL-2^high^ cells in CD8^+^ T cells. Healthy donor PBMCs stimulated with anti-CD3 monoclonal antibody and IL-2 (200 IU/ml) were cultured in medium containing 0.1 µM VEN for 96 hours and then analyzed with flow cytometry (*n* = 3). **E** In vitro killing assay. CellTrace Yellow-labeled KG-1 cells were cocultured with VEN-exposed T cells. Twenty-four hours later, cells were stained with Annexin V and analyzed with flow cytometry (*n* = 3). Unpaired *t*-tests were used for statistical calculations.; *P < 0.05; **P < 0.01.

To verify whether VEN enhances the T-cell anti-tumor immune response, we conducted in vitro experiments using T cells from healthy donor PBMCs. After T cells were exposed to VEN, the proportion of BCL-2^high^ CD8^+^ T cells significantly increased (Fig. 2D). An in vitro killing assay revealed that VEN-exposed T cells exhibited stronger cytotoxicity against the leukemia cell line (Fig. 2E). These results indicate that VEN induces modification of the CD8^+^ T-cell phenotype and subsequent enhancement of its anti-leukemic effects, which can lead to a more substantial “graft-versus-leukemia effect.”

In this study, immunological analyses revealed that in addition to the anticipated preservation of anti-leukemic immunity, BCL-2^high^ CD8^+^ T_EM_ cells induced by VEN therapy exhibited progenitor/non-exhausted, cytotoxic, and tumor-specific capacities, which could mediate a strong “graft-versus-leukemia effect.” Moreover, the clinical analysis showed that VEN therapy improved overall survival and relapse mortality in patients with relapsed AML after allo-HCT, with low non-relapse mortality.

Immunological analyses in this study showed that the number of T cells, in contrast to that of B cells, was relatively maintained during VEN therapy. Notably, BCL-2 expression in CD8^+^ T_EM_ cells increased after VEN therapy, and these cells had progenitor/non-exhausted, cytotoxic, and leukemia-specific capacities. Although the function of BCL-2 in T cells has not been thoroughly clarified, BCL-2 can be upregulated through TCR stimulation and shifted to BCL-XL or MCL-1 to resist mitochondria-related apoptosis following VEN exposure [8, 9]. *BCL-2* gene-transduced chimeric antigen receptor T cells have been reported to highly express *IFN-γ*-related genes and amplify their anti-tumor activities [10]. These results support our theory that the increased number of BCL-2^high^ CD8^+^ T_EM_ cells following VEN therapy can potentiate anti-leukemic immunity.

Still, relapse is the major cause of treatment failure after allo-HCT, and the prognosis is dismal. There are limited therapeutic options, especially early after allo-HCT, with little spare capacity for intensive chemotherapy. Dysfunctional anti-leukemic immune surveillance caused by T-cell exhaustion leads to AML relapse after allo-HCT [11]. Attempts to reinvigorate attenuated T cells by donor lymphocyte infusion (DLI) have been unsuccessful, regardless of the combination with conventional chemotherapy, because cytotoxic agents impair not only leukemia cells but also donor-derived T cells; the potency of the graft-versus-leukemia effect is reduced [12]. VEN therapy has been shown to be highly effective against leukemia cells with only mild toxicity [2]. Several small studies have shown better clinical outcomes in patients with relapsed AML after allo-HCT with VEN combination therapy [13]. In line with these previous studies, our data showed a clinical benefit of VEN therapy relative to well-matched controls defined using propensity score matching.

Regarding the limitations associated with this study, immunomodulatory changes can be induced by DLI or azacytidine. Four of the 12 patients received DLI, and post-treatment parameters were comparable, regardless of DLI (Fig. S3a–c). In addition, patients treated with azacitidine monotherapy showed no immunomodulatory changes (Fig. S4a–c). These interventions seemed to have a limited effect on our findings. The retrospective design, small sample size, and short follow-up period in the VEN therapy group (median, 536 days) were also limitations. Further investigations with larger sample sizes are required to validate our results.

Overall, our study provides a rationale for VEN therapy to enhance anti-leukemic immunity by inducing BCL-2^high^ T cells with progenitor/non-exhausted, cytotoxic, and tumor-specific capacities. We believe that our new finding on BCL-2^high^ CD8^+^ T_EM_ cells can serve as a foundation for the development of novel biomarkers or treatments for refractory AML.

### Supplementary information

Supplementary Figure Legends

Figure S1

Figure S2

Figure S3

Figure S4

Supplementary methods

Supplementary tables

## Data Availability

The data supporting the findings of this study are available from the corresponding author upon reasonable request.
