# Supplementary material for: T cells with high BCL-2 expression induced by venetoclax impact anti-leukemic immunity “graft-versus-leukemia effects”
Source: Blood Cancer J. 2024 May 14;14(1):79. doi: 10.1038/s41408-024-01064-0 (PMC11094022; doi:10.1038/s41408-024-01064-0)
Supplement: Supplementary file 1 — Supplementary Figure Legends [file 41408_2024_1064_MOESM1_ESM.docx]

**Supplementary Figure Legends**

**Figure S1. VEN-therapy shows significantly better clinical outcomes with preservation of CD4^+^ and CD8^+^ T cells in both a propensity score-matched cohort and the entire cohort. (a-c),** Comparison of overall survival (a), relapse mortality (b), and non-relapse mortality (c) between VEN-therapy and control patients in the entire cohort. **(d, e)**, Comparison of relapse mortality (d) and non-relapse mortality (e) between VEN-therapy and control patients in the propensity score-matched cohort. **(f)**, Longitudinal changes in the numbers of CD4^+^ T cells, CD8^+^ T cells, and CD19^+^ B cells in peripheral blood following VEN-therapy. While the numbers of CD4^+^ and CD8^+^ T cells are relatively maintained after VEN-therapy, the number of CD19^+^ B cells decreases.

**Figure S2. Phenotype of T-cells derived from PBMCs of pre- and post-VEN-therapy patients with relapsed AML after allo-HCT. (a),** Representative heatmaps depicting the expression level of each marker and spatial localization on the t-SNE map. **(b),** BCL-2 expression of CD8^+^ T cells was analyzed before and after VEN-therapy. Representative data are shown. **(c-e),** Proportions of PD-1^high^ (c), IFN-γ^+^GZMB^+^ (d), and WT-1 tetramer^+^ (e) cells in whole CD8^+^ T cells are shown. Flow cytometry analysis was performed on PBMCs for PD-1 and WT-1 data, and on bone marrow samples for cytokine data.

**Figure S3. Effects of additional donor lymphocyte infusion on immune phenotypic changes in CD8^+^ T-cells derived from PBMCs of VEN-therapy patients with relapsed AML after allo-HCT. (a-c),** A comparison of immunophenotype of CD8^+^ T cells from post-VEN-therapy patients with or without DLI. The BCL-2 expression (n=11) (a), proportions of PD-1^high^ (n=11) (b), and IFN-γ^+^GZMB^+^ (n=8) (c) in CD8^+^ T cells were evaluated. PBMCs from patients were analyzed with flow cytometry. Unpaired *t*-tests were used for statistical analyses. ns, not significant.

**Figure S4. Effects of azacitidine monotherapy on immune phenotypic changes in CD8^+^ T-cells derived from PBMCs of patients with relapsed myelodysplastic syndrome after allo-HCT. (a-c)**, A comparison of immunophenotype of CD8^+^ T cells from pre- and post- azacitidine monotherapy patients. The BCL-2 expression (a), proportions of PD-1^high^ (b), and IFN-γ^+^GZMB^+^ (c) in CD8^+^ T cells were evaluated. PBMCs from patients were analyzed with flow cytometry (n=3). A paired two-tailed Student’s *t*-test was utilized for statistical analyses. ns, not significant.
