# Supplementary figures and images for: T cells with high BCL-2 expression induced by venetoclax impact anti-leukemic immunity “graft-versus-leukemia effects”
Source: Blood Cancer J. 2024 May 14;14(1):79. doi: 10.1038/s41408-024-01064-0 (PMC11094022; doi:10.1038/s41408-024-01064-0)

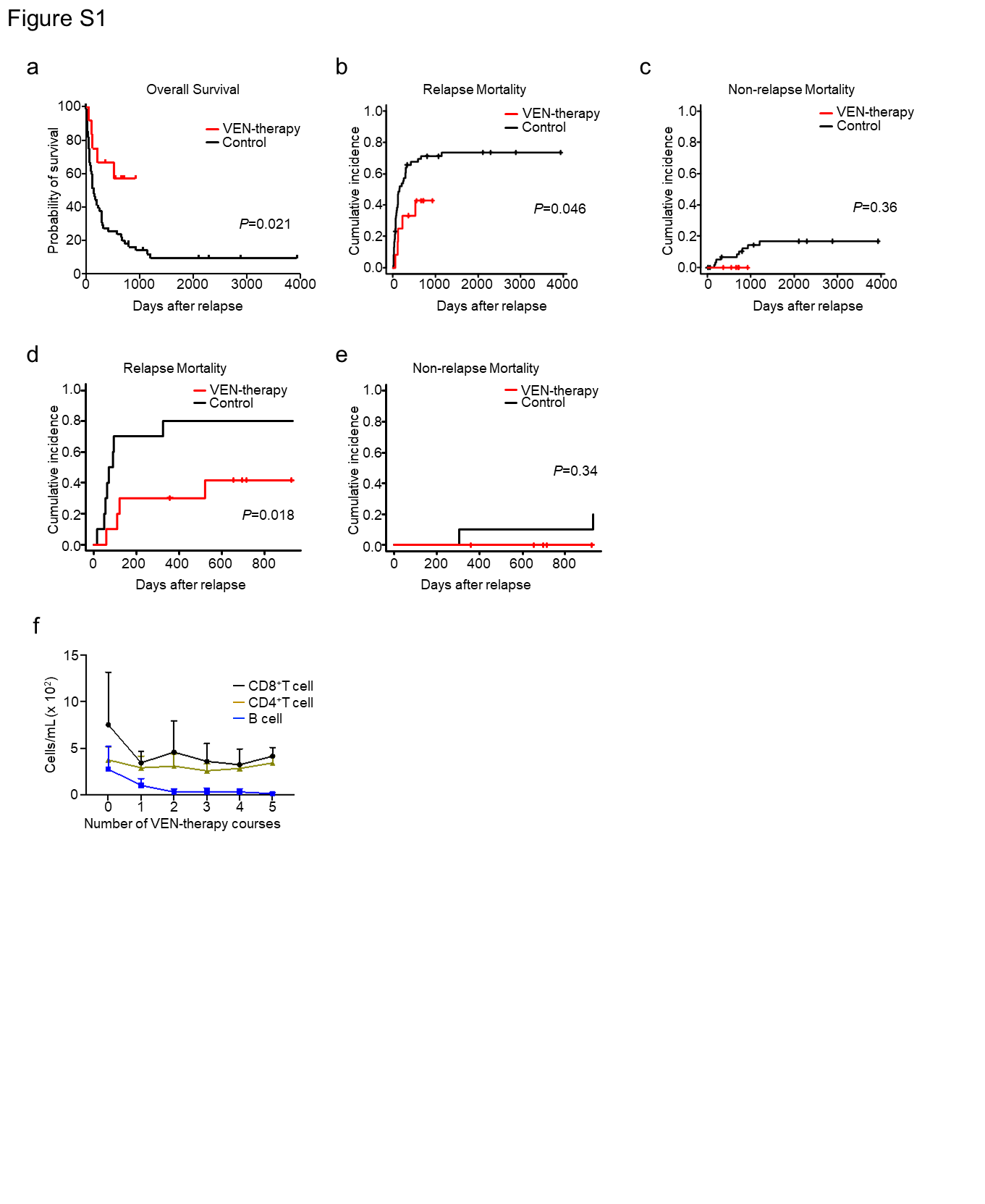

Supplement: Supplementary file 2 — Figure S1 [file 41408_2024_1064_MOESM2_ESM.tif]

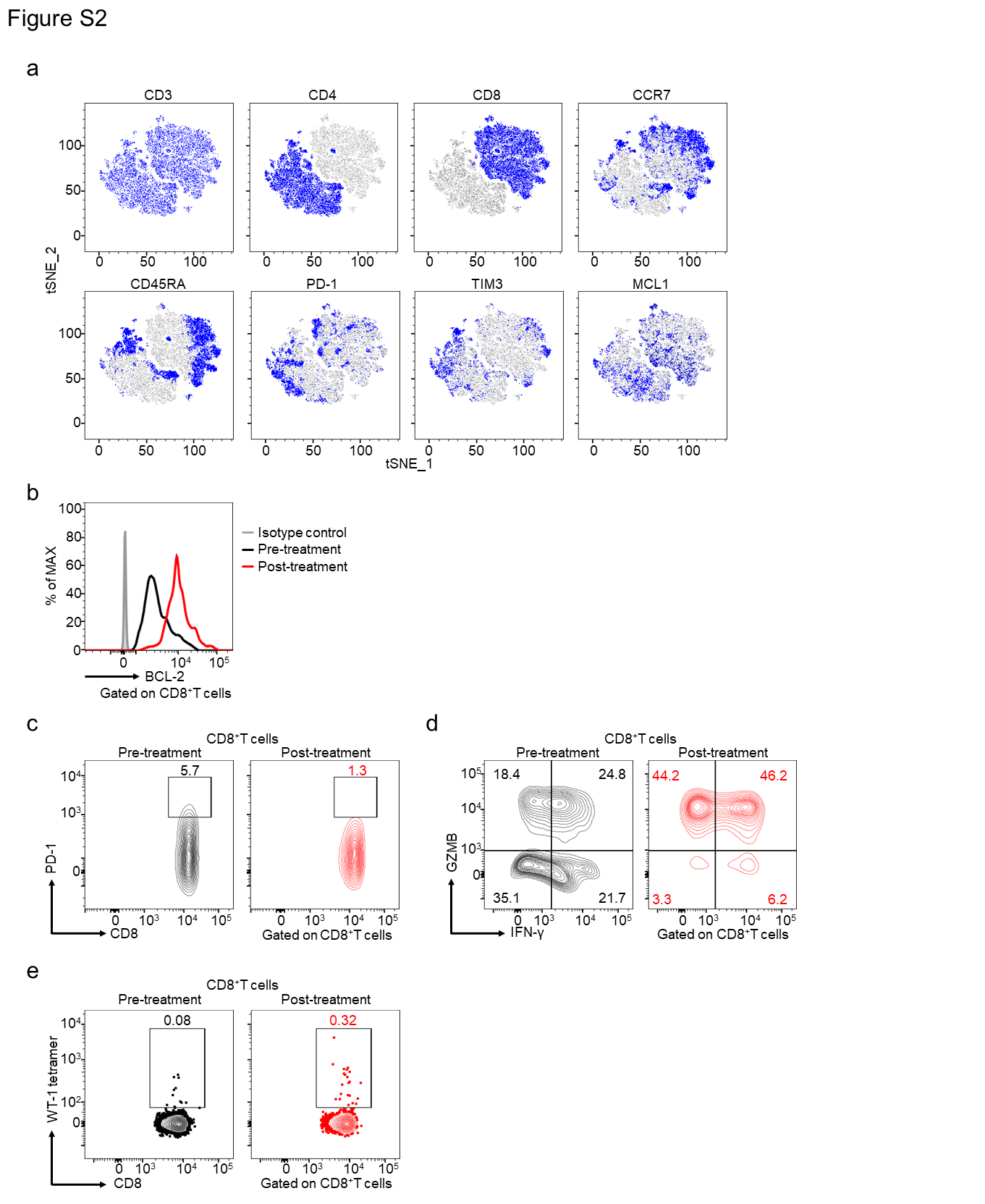

Supplement: Supplementary file 3 — Figure S2 [file 41408_2024_1064_MOESM3_ESM.tif]

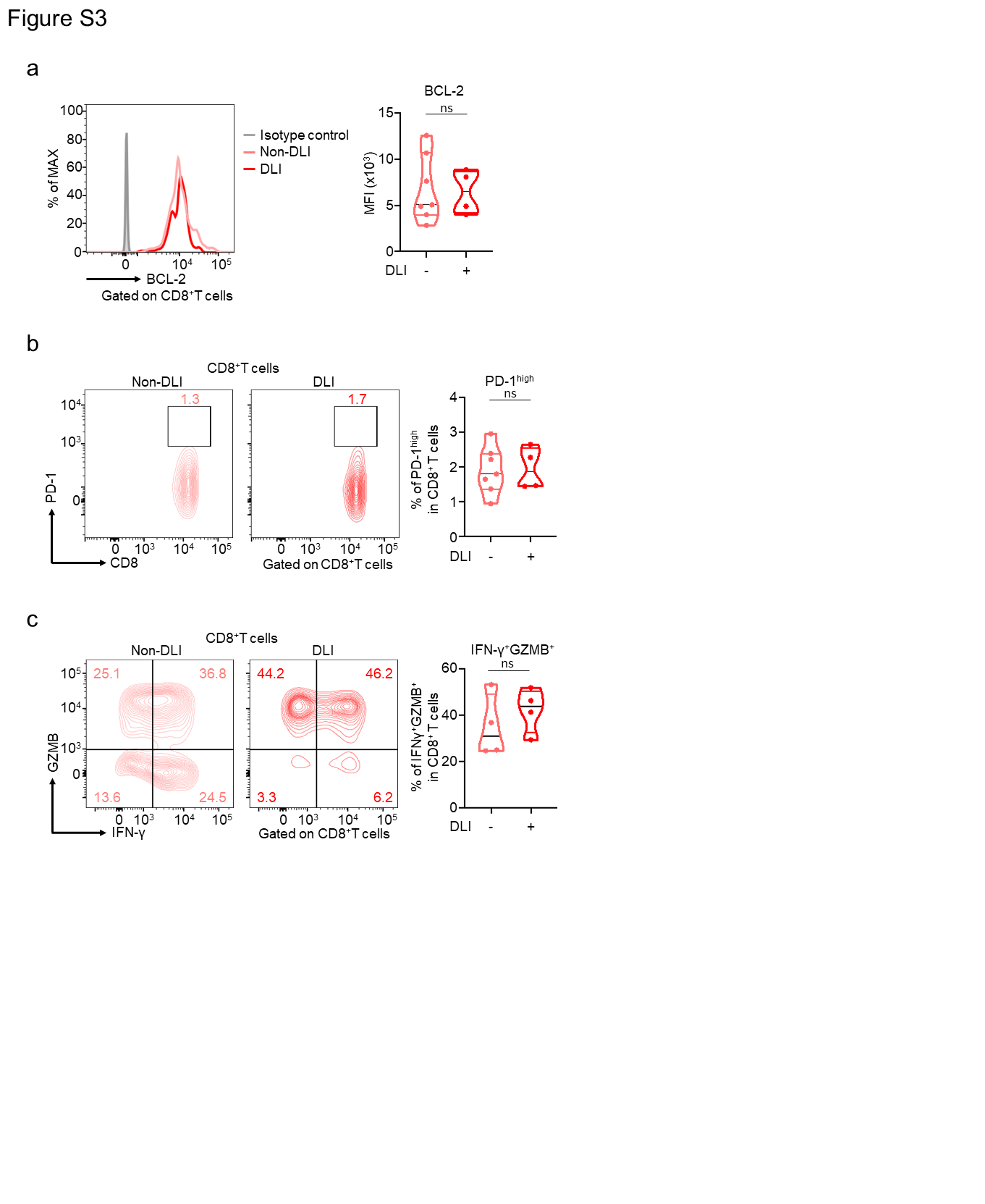

Supplement: Supplementary file 4 — Figure S3 [file 41408_2024_1064_MOESM4_ESM.tif]

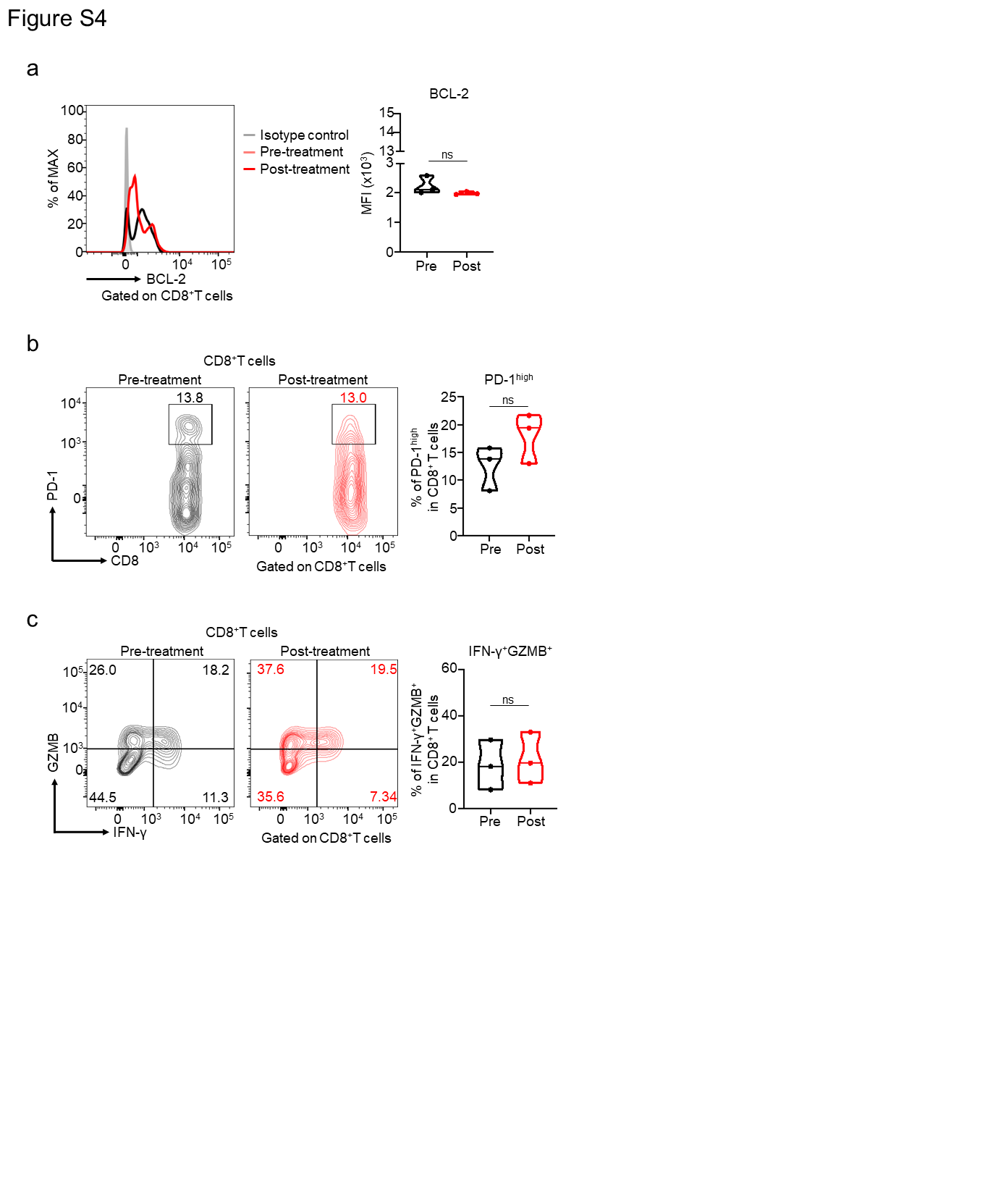

Supplement: Supplementary file 5 — Figure S4 [file 41408_2024_1064_MOESM5_ESM.tif]
