# Supplementary material for: T cells with high BCL-2 expression induced by venetoclax impact anti-leukemic immunity “graft-versus-leukemia effects”
Source: Blood Cancer J. 2024 May 14;14(1):79. doi: 10.1038/s41408-024-01064-0 (PMC11094022; doi:10.1038/s41408-024-01064-0)
Supplement: Supplementary file 6 — Supplementary methods [file 41408_2024_1064_MOESM6_ESM.docx]

**Supplementary methods**

**Patients and samples**

Patients aged >16 years with hematological or molecular relapsed acute myeloid leukemia (AML) after allogeneic hematopoietic cell transplantation (allo-HCT) who received venetoclax and azacitidine (VEN-therapy) at Osaka Metropolitan University Hospital between January 2021 and December 2022 were enrolled in this study. We selected the control patients with relapsed AML after allo-HCT who were treated at our institution with any therapy other than VEN between January 2010 and December 2022. Hematological relapse was defined as bone marrow blasts >5%, appearance of blasts in peripheral blood, or extramedullary disease manifestation. Molecular relapse was defined as recurrence of patient-specific disease markers such as chromosomal aberrations, molecular alterations, or elevations of Wilms’ tumor-1 (WT-1) mRNA at two consecutive time points without evidence of hematologic relapse. Genetic risk was classified using the 2022 ELN risk classification[1]. The criteria concerning the response to VEN-therapy were defined according to the 2022 ELN recommendations[1]. Analysis of clinical information and immune cells in peripheral blood mononuclear cells (PBMCs) or bone marrow fluids was performed. Patients’ clinical information was obtained from their medical records. Clinical samples before and after VEN-therapy were prospectively collected from peripheral blood or bone marrow of patients who gave written informed consent. Mononuclear cells were obtained by gradient centrifugation with a Ficoll-PaqueTM Plus (Cytiva, Tokyo, Japan, Cat# 17-1440-02) at 400 x g at 20°C for 40 minutes, and frozen with BAMBANKER cell-freezing medium (GC Lymphotec, Tokyo, Japan, Cat# CS-02-001) in liquid nitrogen until measurement. PBMCs and bone marrow samples after one or two cycles of VEN-therapy were used for the post-treatment analysis. The protocols were approved by the appropriate institutional review boards and ethics committees at Osaka Metropolitan University Graduate School of Medicine. This study was conducted in accordance with the principles of the Declaration of Helsinki.

**Treatment schedule**

All patients in the VEN-therapy group received azacitidine 75 mg/m^2^/day for 5 consecutive days concurrently with venetoclax (VEN). In principle, the first course consisted of VEN administration for 14 days to reduce the risk of severe myelosuppression. In the second course and later, VEN was administered for 14 to 28 days depending on the severity of toxicities during the course. The VEN dose was ramped up from 50 mg to 200 mg if given concurrently with fluconazole, or from 25 mg to 50 mg if given concurrently with itraconazole, considering drug-interaction. In principle, donor lymphocyte infusion was started on between days 7 and 28 in the first course of VEN-therapy if available.

**Cell lines**

The KG-1 cell line (human leukemia, RRID: CVCL_0374, Cat#RCB1166) was provided by the RIKEN BRC through the National Bio Resource Project of the MEXT, Japan. This cell line was used after confirming that the cells were *Mycoplasma* (-) using the PCR Mycoplasma Detection Kit (Takara Bio Inc. Shiga, Japan, Cat#6601), according to the manufacturer’s instructions.

**Flow cytometry analyses**

Flow cytometry assays were performed as described previously[2]. Briefly, the cells were thawed and washed with phosphate-buffered saline containing 2% fetal bovine serum (FBS) and stained with surface antibodies. Intracellular staining was performed with specific antibodies and the FOXP3/Transcription Factor Staining Buffer set (Thermo Fisher Scientific, Waltham, MA, US, Cat#00-5523-00), according to the manufacturer’s instructions. Cells were stained with the following fluorophore-conjugated antibodies: MCL1 Alexa Fluor 488 (Abcam, Cambridge, United Kingdom, clone Y37, Cat#ab197529, 1/40000), Granzyme B FITC (BioLegend, San Diego, CA, clone GB11, RRID: AB_2114575, Cat# 515403, 1/100), Interferon-γ PerCP/Cyanine5.5 (BioLegend, clone 4S.B3, RRID:AB_961357, Cat# 502525, 1/200), PD-1 APC (BioLegend, clone EH12.2H7, RRID:AB_940488, Cat# 329902, 1/200), CD3 Alexa Fluor 700 (BioLegend, clone UCHT1, RRID:AB_ 493740, Cat# 300423, 1/200), BCL-2 BV421 (BioLegend, clone 100, RRID:AB_2563283, Cat# 658709, 1/100), CCR7 BV605 (BioLegend, clone G043H7, RRID:AB_2561753, Cat# 353224, 1/100), CD45RA Brilliant Violet 711 (BioLegend, clone HI100, RRID:AB_2563815, Cat# 304138, 1/100), TIM-3 PE-Cy7 (Biolegend, clone F38-2E2, RRID:AB_ 2561720, Cat# 345014, 1/200), CD4 BUV395 (Becton Dickinson and Company, Franklin Lakes, NJ, US, clone RPA-T4, RRID:AB_2738917, Cat#564724, 1/100), CD8 PE/Cyanine7 (BioLegend, clone SK1, RRID:AB_2044008, Cat#344712, 1/100), and CD8 BUV496 (Becton Dickinson and Company, clone RPA-T8, RRID:AB_2870223, Cat#612942, 1/100). Dead cells were excluded using Fixable Viability Dye eFluor 780 (eBioscience, Waltham, MA, US, Cat#65-0865-14). Using WT-1 peptide/HLA-A*24:02 tetramer (MBL, Tokyo, Japan, Cat#TS-M014-1), the frequencies of WT1 TCR-positive T cells were analyzed by flow cytometry. For intracellular cytokine staining, cells were stimulated for 5 hours with phorbol 12-myristate 13-acetate (Sigma Aldrich, St. Louis, MO, US, Cat#P8139-1MG, 100 ng/mL) and ionomycin (Sigma Aldrich, Cat#I0634-1MG, 2 μg/mL) in the presence of GolgiPLUG reagent (BD Biosciences, RRID: AB_2869014, Cat#555029, 1.3 μl/ml). Staining antibodies were diluted according to the manufacturer’s instructions. Samples were assessed with a BD LSRFortessa X-20 Cell Analyzer (BD Biosciences, RRID:SCR_019600) and FlowJo software ver.10 (BD Biosciences, RRID:SCR_008520). For t-SNE analysis, data files were passed through a preprocessing pipeline that included cleanup for viability, cell aggregates, and instrument acquisition anomalies using a combination of manual gating and the flowAI plug-in (FlowJo Exchange). Files were downsampled to a fixed number of lymphocytes after gating for Live/Singlet/CD3^+^ events per sample. Downsampled events were concatenated into a single file, and the t-SNE algorithm was applied using all antibodies in the panel as parameter input values. t-SNE X and Y parameters were plotted for the fully concatenated file (Fig. 1c) or the deconvolved, pre- and post-treatment group files (Fig. 1d) to assess global changes in population frequencies. Visible populations based on the t-SNE plot were manually gated to explore relevant subsets and the expression of additional markers on these subsets.

***In vitro* expansion of T cells with VEN reagent**

Healthy donor PBMCs were plated into a 96-well plate and cultured with RPMI1640 including 10% FBS, IL-2 (PeproTech, London, England, Cat#PEP-200-02-1, 200 IU/mL), anti-CD3 monoclonal antibody (eBiscience, RRID: AB_468855, Cat#16-0037-85, clone OKT-3, 0.2 µg/mL), and venetoclax (Toronto Research Chemicals Inc, Toronto, ON, Canada, Cat#A112425, 0.1 nM) at 37 °C. After 96 hours, expanded T cells were harvested and stained for flow cytometry analysis or used for the killing assay.

**Killing assay**

Killing assays were performed with the modified method using FITC-Annexin V (BioLegend, Cat#640914) and CellTrace Yellow (Thermo Fisher Scientific, Cat#C34573) as previously reported[3]. Briefly, T cells were expanded from the healthy donor PBMCs in the manner described above. Then, CellTrace Yellow-labeled tumor cells (KG-1) were cocultured with these VEN-exposed T cells at a 1:1 ratio and mixed well to ensure contact between the cell populations. After 24 hours of incubation, flow cytometry analysis was performed. *In vitro* experiments were performed in triplicate.

**Statistical analyses**

For clinical statistics, to perform the matched-pair analysis between VEN-therapy and control patients, we first selected the following independent covariates prior to treatment assignment that could relate significantly to the clinical outcomes: age, sex, time from relapse to transplantation, disease status at relapse, karyotype risk, and blasts in the bone marrow at the start of treatment. Then, multivariable logistic regression analysis was used to determine the probability of VEN-therapy, with the propensity score ranging from 0 to 1, for each patient in the data set. We performed 1:1 caliper matching using the nearest-neighbor matching method, with a caliper width fixed at 0.2 of the standard deviation of the propensity score. The covariate balances were checked using a receiver operating characteristic curve analysis of the area under the curve of the propensity score. The standardized differences between two groups were also assessed. Patient characteristics were compared between the two groups using the Fisher exact test for categorical covariates and the Mann-Whitney U test for continuous covariates. Overall survival was defined as the time from the relapse of AML after allo-HCT until death from any cause. Relapse mortality and non-relapse mortality were defined as the time from the relapse of AML after allo-HCT until death from AML or any cause other than AML, respectively. Overall survival was calculated using the Kaplan-Meier method and compared statistically between groups using the log-rank test. Cumulative incidence curves were used to analyze relapse mortality and non-relapse mortality to accommodate competing risks. Relapse mortality and non-relapse mortality were mutually competing. Gray’s test was used to compare cumulative incidences. The therapeutic impact of VEN-therapy, compared with control, was estimated using the Cox proportional hazards model for overall survival. For experimental statistics, the unpaired two-tailed Student’s t-test was applied for comparison between two groups. Wilcoxon signed rank test was used to analyze related samples. Data are depicted as mean ± standard error (error bars). *P*-values < 0.05 were considered statistically significant. All statistical analyses and generation of graphs were performed using EZR version 1.37 (Saitama Medical Center, Jichi Medical University, Saitama, Japan) and GraphPad Prism 9 (GraphPad Software, La Jolla, CA, US).

References

1. Döhner H, Wei A, Appelbaum F, Craddock C, DiNardo C, Dombret H*, et al.* Diagnosis and management of AML in adults: 2022 recommendations from an international expert panel on behalf of the ELN. *Blood* 2022; 140**:** 1345-1377.

2. Nagasaki J, Togashi Y, Sugawara T, Itami M, Yamauchi N, Yuda J*, et al.* The critical role of CD4+ T cells in PD-1 blockade against MHC-II-expressing tumors such as classic Hodgkin lymphoma. *Blood Adv* 2020; 4**:** 4069-4082.

3. Pourzia AL, Olson ML, Bailey SR, Boroughs A, Aryal A, Ryan J, Maus MV, Letai A. Quantifying requirements for mitochondrial apoptosis in CAR T killing of cancer cells. Cell Death Dis. 2023 Apr 13;14(4):267. doi: 10.1038/s41419-023-05727-x.
