# Supplementary material for: T cells with high BCL-2 expression induced by venetoclax impact anti-leukemic immunity “graft-versus-leukemia effects”
Source: Blood Cancer J. 2024 May 14;14(1):79. doi: 10.1038/s41408-024-01064-0 (PMC11094022; doi:10.1038/s41408-024-01064-0)
Supplement: Supplementary file 7 — Supplementary tables [file 41408_2024_1064_MOESM7_ESM.docx]

Table S1. Characteristics of patients receiving VEN-therapy

| ID | Sex | Age | Prior MDS | Source of first Tx | FAB classification | Cytogenetic/molecular abnormalities  at diagnosis | | Days of relapse from first Tx | Best response to VEN-therapy | Days of best response from VEN-therapy | Second Tx | DLI |
| --- | --- | --- | --- | --- | --- | --- | --- | --- | --- | --- | --- | --- |
| N001 | m | 41 | No | BM from MUD | M4 | t(11;19)(q23.3;p13.1) | KMT2A-ELL | 91 | CR _MRD-_ | 247 | Yes | Yes |
| N002 | f | 53 | No | BM from MUD | M5b | Normal karyotype | KMT2A-AFDN | 175 | CR _MRD-_ | 19 | No | Yes |
| N003 | f | 61 | No | BM from MUD | M0 | Normal karyotype |  | 183 | CR _MRD-_ | 17 | No | Yes |
| N004 | m | 69 | No | PB from MMRD | M2 | Complex karyotype |  | 185 | CR _MRD-_ | 34 | Yes | No |
| N005 | f | 50 | No | PB from MaRD | M5a | Normal karyotype |  | 890 | CR _MRD-_ | 31 | No | Yes |
| N006 | m | 66 | Yes | PB from MMRD | M5b | del(13)(q?), inv(6)(p23q15) | FLT3-ITD | 147 | No response | - | No | No |
| N008 | f | 59 | Yes | Cord blood | M2 | Complex karyotype |  | 1047 | CR _MRD+_ | 73 | Yes | No |
| N010 | f | 47 | No | PB from MUD | M2 | Complex karyotype |  | 1201 | CR _MRD-_ | 41 | Yes | No |
| N011 | f | 49 | Yes | Cord blood | M2 | Complex karyotype |  | 1092 | CRi _MRD+_ | 161 | Yes | No |
| N013 | m | 50 | Yes | PB from MMRD | M2 | Normal karyotype |  | 1567 | CR _MRD+_ | 47 | Yes | No |
| N015 | m | 60 | Yes | PB from MMRD | M5b | Complex karyotype |  | 120 | No response | - | No | No |
| N018 | f | 65 | Yes | PB from MUD | M4 | t(1;10)(p22;p11.2), t(1:9)(p13:q34.1) | FLT3-ITD | 75 | No response | - | No | No |

Abbreviations: VEN, venetoclax; BM, bone marrow; PB, peripheral blood; MaRD, matched related donor; MUD, matched unrelated donor; MMRD, mismatched related donor; CR _MRD-_, complete remission without minimal residual disease; CR _MRD+_, complete remission with minimal residual disease; CRi, complete remission with incomplete hematologic recovery; Tx, transplantation; DLI, donor lymphocyte infusion

Table S2. Patient characteristics before and after propensity score matching

| Parameters | | | | Before propensity score matching | | |  | After propensity score matching | | |  |
| --- | --- | --- | --- | --- | --- | --- | --- | --- | --- | --- | --- |
|  |  |  |  | VEN-therapy  (N=12) | Control  (N=61) | *P* value |  | VEN-therapy  (N=10) | Control  (N=10) | *P* value | Standardized difference |
| Age, years | | | | 56 (41-69) | 50 (18-69) | 0.04 |  | 57 (41-69) | 62 (43-68) | 0.71 | 0.166 |
| Male | | | | 5 (42%) | 36 (59%) | 0.35 |  | 4 (40%) | 5 (50%) | 1.00 | 0.202 |
| Disease status at relapse | | | |  |  | 0.25 |  |  |  | 1.00 | <0.001 |
|  | | Molecular relapse | | 2 (17%) | 4 (7%) |  |  | 2 (20%) | 2 (20%) |  |  |
|  | | Non-molecular relapse | | 10 (83%) | 57 (93%) |  |  | 8 (80%) | 8 (80%) |  |  |
| Karyotype risk | | | |  |  | 1.00 |  |  |  | 1.00 | 0.202 |
|  | | | Favorable | 0 (0%) | 3 (5%) |  |  | 0 (0%) | 0 (0%) |  |  |
|  | | | Intermediate | 6 (50%) | 29 (48%) |  |  | 4 (40%) | 5 (50%) |  |  |
|  | | | Adverse | 6 (50%) | 29 (48%) |  |  | 6 (60%) | 5 (50%) |  |  |
| Days of relapse from first transplantation | | | | 184 (75-1567) | 135 (14-2154) | 0.06 |  | 179 (75-1251) | 179 (19-2154) | 0.71 | 0.043 |
| Blasts in BM at start of treatment | | | |  |  |  |  |  |  | 0.485 | 0.535 |
|  | < 5% | | | 4 (33%) | 20 (33%) |  |  | 4 (40%) | 4 (40%) |  |  |
|  | 5 to 20% | | | 2 (17%) | 19 (31%) |  |  | 2 (20%) | 4 (40%) |  |  |
|  | > 20% | | | 6 (50%) | 22 (36%) |  |  | 4 (40%) | 2 (20%) |  |  |

Abbreviations: VEN, venetoclax; BM, bone marrow
